# Description of a novel RyR2 mutation in a juvenile patient with symptomatic catecholaminergic polymorphic ventricular tachycardia in sleep and during exercise: a case report

**DOI:** 10.1186/s13256-018-1825-6

**Published:** 2018-10-09

**Authors:** L. K. Seidlmayer, F. Riediger, N. Pagonas, P. Nordbeck, O. Ritter, B. Sasko

**Affiliations:** 1 Internal Medicine 1, Department of Cardiology, University Hospital of Wurzburg, Oberduerrbacherstrasse 6, 97080 Wurzburg, Germany; 2 Comprehensive Heart Failure Center, Am Schwarzenberg 15, 97078 Wurzburg, Germany; 3 Department of Cardiology, Brandenburg Medical School Brandenburg – Theodor Fontane (MHB), University Hospital Brandenburg, Hochstrasse 29, 14470 Brandenburg an der Havel, Germany

**Keywords:** Catecholaminergic polymorphic ventricular tachycardia, CPVT, Ryanodine receptor type 2 (RyR2), Novel gene mutation, Cardiac arrest

## Abstract

**Background:**

Catecholaminergic polymorphic ventricular tachycardia is an inherited disease presenting with arrhythmic events during physical exercise or emotional stress. If untreated, catecholaminergic polymorphic ventricular tachycardia is a highly lethal condition: About 80% of affected individuals experience recurrent syncope, and 30% experience cardiac arrest. Catecholaminergic polymorphic ventricular tachycardia is caused by mutations in genes encoding ryanodine receptor type 2 (RyR2) and cardiac calsequestrin (CASQ2). In cases of sympathoadrenergic activation, both mutations result in a spontaneous Ca^2+^ release in cardiac cells, facilitating ventricular arrhythmias.

**Case presentation:**

We present a case of a 17-year-old Caucasian boy who survived sudden cardiac death caused by ventricular fibrillation while performing running exercise in a fitness center. The diagnostic workup included blood tests, coronary angiography, electrophysiological testing, and cardiac magnetic resonance imaging, but all results were normal. Because the patient’s medical history included recurrent syncope during physical and emotional stress, we strongly suspected catecholaminergic polymorphic ventricular tachycardia as the underlying disease. Genetic screening was performed and confirmed the diagnosis, revealing a new heterozygous point mutation in the gene for RyR2, c.12520T>A (p.F4174 l, exon 90, RyR2 gene). The patient was discharged from our hospital after undergoing implantation of an implantable cardioverter defibrillator for secondary prevention. Shortly after implantation, the implantable cardioverter defibrillator terminated a sustaining ventricular tachycardia episode by antitachycardic pacing. This episode occurred early in the morning while the patient was asleep.

**Conclusions:**

We present a case of catecholaminergic polymorphic ventricular tachycardia associated with a novel single point mutation in the RyR2 gene, which, to the best of our knowledge, has not been described in the literature so far. Our patient experienced arrhythmic events under both resting conditions and physical activity, an uncommon finding in patients with catecholaminergic polymorphic ventricular tachycardia. This novel mutation may cause arrhythmias independent of sympathoadrenergic stimulation, but further evidence is needed to prove causality.

## Background

Catecholaminergic polymorphic ventricular tachycardia (CPVT) is an inherited disease presenting with recurrent syncopes during exercise and acute emotions. The prevalence of CPVT in Europe ranges from 1 to 5 in 10,000 individuals [1]. The first symptoms usually occur during childhood or young adolescence. If untreated, CPVT is a highly lethal condition: About 30% of affected individuals experience cardiac arrest, and 80% experience recurrent syncopes [2]. Affected individuals usually present without any structural cardiac abnormality and show normal electrocardiogram (ECG) findings under resting conditions. In case of sympathoadrenergic activation, the underlying ryanodine receptor 2 (RyR2) and calsequestrin (CASQ2) mutations result in polymorphic or bidirectional ventricular tachycardia (VT) [3]. Suggested treatment strategies include beta-blockers, flecainide, sympathic denervation, and implantable cardioverter defibrillators (ICDs) [4]. However, despite these treatment options, physical exercise should be limited to avoid sympathoadrenergic stimulation and the provocation of arrhythmic events. This case report describes a novel single point mutation in the RyR2 gene, which has not been described before. Additionally, this mutation may cause arrhythmias independent of sympathoadrenergic stimulation.

## Case presentation

**Fig. 1:**
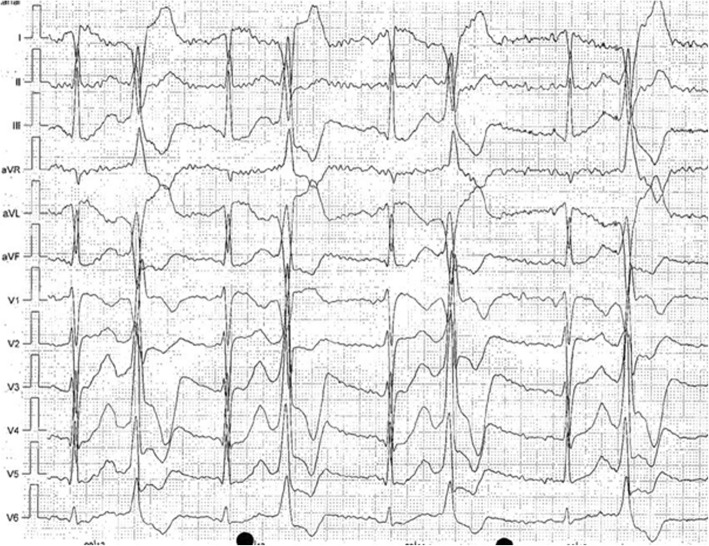
Twelve-lead electrocardiogram obtained during ergometry showing a bigeminus with right bundle branch block morphology at 80 W. At this point, ergometry was stopped because recurrent syncopes under physical activity were reported in this patient

A 17-year-old Caucasian boy was admitted to our intensive care unit (ICU) after successful resuscitation by emergency services. While performing running exercise in a fitness center, he suddenly collapsed. Because neither pulse nor breathing could be detected by the bystanders, immediate resuscitation was performed. In the first heart rhythm analysis conducted by the paramedics, ventricular fibrillation (VF) was seen and immediately defibrillated into sinus rhythm. The patient recovered quickly and was transferred to our ICU by the ambulance service. At admission, the patient was in hemodynamically stable condition with normal vital signs (heart rate 95/min, blood pressure 125/79 mmHg, auricular temperature 36.5 °C, respiration 15 breaths/min, oxygen saturation of 100% on 4-L nasal cannula). The physical examination revealed no abnormal findings. Auscultation of the heart showed a regular rate and rhythm with normal S1 and S2 and no murmurs or rubs. The breath sounds of the lungs were equal and clear bilaterally with no wheezes, rhonchi, or rales. The patient was awake (Glasgow Coma Scale score of 15) and orientated in all aspects. No focal sensory or motor deficits, aphasia, or inadequate balances were noted in the neurological examination. Deep tendon reflexes and cranial nerves II through XII were intact. Because there were no cerebral or other sequelae at the time of hospital admission, we decided not to obtain a cranial computed tomographic scan, owing to the patient’s young age. When asked about the event, he told us that he had no symptoms prior to the collapse. However, in the years before, he had syncopated several times while climbing stairs, playing soccer, and once when he got frightened. A general practitioner previously performed an exercise ECG, which showed multiple premature beats under submaximal stress (Fig. 1). As a result, beta-blockers were prescribed (metoprolol succinate 47.5 mg once per day). Apart from this, the patient had no medical history or prior medication. The patient was a nonsmoker with no regular alcohol consumption and an unremarkable family, social, and environmental history.

**Fig. 2:**
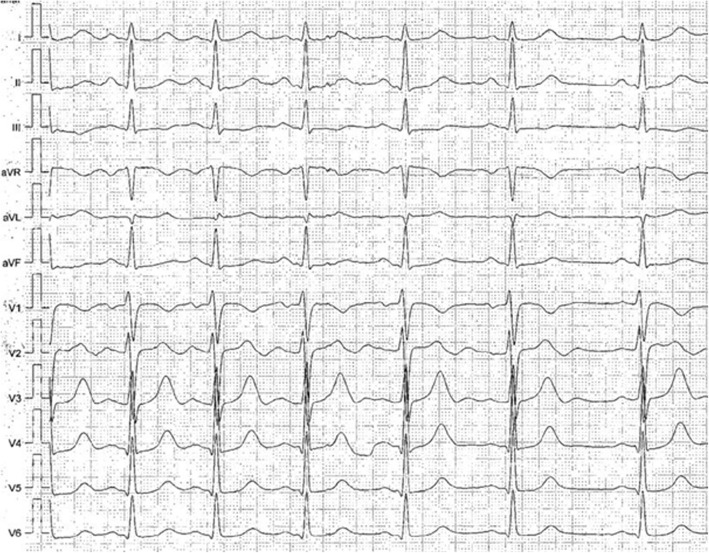
Resting 12-lead electrocardiogram of the described patient showing negative T-waves in V1 as well as biphasic T-waves in V2

At ICU admission, initial medical treatment included the first surface ECG after cardiac arrest, which showed negative T-waves in V1 and biphasic T-waves in V2 (Fig. 2). The results of blood tests showed normal findings without signs of cardiac ischemia or metabolic disorders (Table 1). Respiratory disorders (bronchospasm, aspiration) or neurovascular events seemed to be unlikely because the physical examination, blood gas analysis, and a pulmonary x-ray showed normal findings. An echocardiogram revealed normal left ventricular function without wall motion abnormalities, right heart strain, or valve disease. Additionally, a cardiac magnetic resonance imaging scan was performed and showed normal findings. Therefore, we ruled out hypertrophic and dilative cardiomyopathy, arrhythmogenic right ventricular cardiomyopathy, and acute myocarditis as potential differential diagnoses. Further diagnostic workup included coronary angiography and electrophysiological testing, but none of those resulted in any relevant finding.

**Table 1:** Summary of laboratory test results

Laboratory test	Result
Hemoglobin, g/dl	15.4
White blood cell, × 10^9^/L	10.9
Platelets, × 10^9^/L	354
High-sensitivity troponin T, pg/ml	12
D-dimer, mg/dl	457
NT-proBNP, pg/ml	102
CRP, mg/dl	0.4
Serum sodium, mmol/L	141
Serum potassium, mmol/L	4.7
Serum creatinine, mg/dl	1.17
INR	1.1
PTT, s	34.7
LDH, U/L	201
GOT, U/L	32.6
GPT, U/L	32.1
Serum bilirubin total, μmol/L	14.2
Serum glucose, mg/dl	128

*Abbreviations: NT-proBNP* N-terminal prohormone of brain natriuretic peptide, *CRP* C-reactive protein; *INR* International normalized ratio, *PTT* Partial thromboplastin time, *LDH* Lactate dehydrogenase, *GOT* Aspartate aminotransferase, *GPT* Alanine aminotransferase

Because no extracardiac cause or structural heart disease was found, diagnostic workup focused on channelopathies and genetic heart diseases. Owing to the patient’s medical history of stress-dependent syncope, we strongly suspected a CPVT as the underlying mechanism for VF. Genetic screening was performed and confirmed the diagnosis of CPVT, revealing a new heterozygous point mutation in the gene for ryanodine receptor type 2, which, to the best of our knowledge, has not been described to date in the literature. The heterozygous mutation c.12520T>A (p.F4174 l, exon 90, RyR2 gene) results in an exchange of phenylalanine to isoleucine at position 4174 of the RyR2 protein. Forty-five percent of the relevant mutations of RyR are located in this region [1]. This novel identified missense mutation is located in the C-terminal channel region as a residue of the hydrophobic side chain of the S6/U motif interface of RyR2 [5].

Prediction algorithms for forecasting functional effects of a mutation clearly identify this mutation to potentially cause CPVT [2]. Genetic screening of both parents showed that neither of them were carriers of the mutation. Results of further screening of the patient and family members for other pathogenic mutations (long or short QT syndrome, Brugada syndrome) were also negative.

On the basis of of our findings, the patient received an ICD device to protect him from recurrent episodes of VF. Additionally, oral medication with a beta-blocker was continued with the maximum tolerable dose. Because of the relatively small number of documented arrhythmic episodes, we did not add flecainide at that point. Left ventricular sympathetic denervation was discussed with the patient and the family, but in the end it was deferred.

**Fig. 3:**
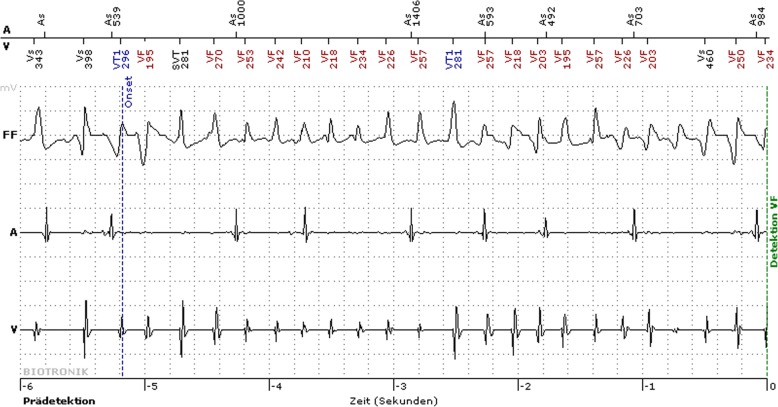
Intracardiac electrogram obtained from implantable cardioverter defibrillator Holter monitor showing a polymorphic ventricular tachycardia during sleep that was terminated by antitachycardic pacing

At hospital discharge, the patient was included in our home monitoring program for ICD surveillance. Shortly after implantation, the ICD terminated a sustaining VT by antitachycardic pacing (Fig. 3). This episode occurred early in the morning while the patient was asleep, which is an uncommon situation for arrhythmic events in patients with CPVT. However, the patient had no memory of a possible emotional stress due to dreams. In the following 6 months, a single episode of VF occurred during a physical activity (cycling), this time terminating spontaneously.

## Discussion

We report a novel single point mutation in the ryanodine receptor (F4174 l) of a juvenile patient with CPVT that possibly also caused VT at rest. This patient harboring the mutation experienced multiple syncopes during exercise or emotional stress and also, unlike other typical CPVT-associated RyR2 mutations, had a VT episode during sleep.

Several studies showed that various mutations in RyR2 account for approximately 50% of all CPVT cases [1]. In this novel mutation, the single-nucleotide substitution T to A results in a conservative amino acid mutation in the C-terminal channel region. We suppose that the substitution could affect the opening and closing features of the S6 helix bundle, possibly resulting in a leaky ion channel and the occurrence of cardiac arrhythmias under adrenergic stimulation. Interestingly, several mutations have been identified in the C-terminal region of this protein, all of them associated with CPVT [6]. Therefore, this specific region seems to represent a vulnerable protein structure with severe clinical impact if affected by gene mutations. Other rare mutations in CPVT include CASQ2, trans-2,3-enoyl-coenzyme A reductase-like protein, calmodulin, and triadin [3].

In experimental settings, most of the CPVT causing mutations in the RyR2 gene result in an increased Ca^2+^ leak from the sarcoplasmic reticulum (SR), which is mediated by the phosphorylation of RyR2 by PKA (protein kinase A). The stimulation of the beta-adrenergic receptor signaling pathway seems to be the most important mechanism for the activation of the PKA-dependent phosphorylation of RyR2. Therefore, adrenergic stimulation during physical or emotional challenges increases the spontaneous Ca^2+^ release from the SR in cardiac cells and facilitates ventricular arrhythmias in affected patient groups. However, previously described CPVT-associated RyR2 mutants, including RyR2-G230C, RyR2-S2226L, RyR2-P2328S, RyR2-R2474S, RyR2-Q4201R, RyR2-R4497C, and RyR2-V4653F, are linked to a PKA-dependent phosphorylation of RyR2 channel activity during sympathoadrenergic stimulation [7–9].

A RyR2-S4565R mutation that has been linked to sudden infant death syndrome has been investigated by analyzing Ca^2+^ release channel activity *in vitro*. In that setting, an increased release of cytosolic Ca^2+^ under basal conditions without PKA treatment has been observed. This suggests the existence of a mutant channel that is more sensitive to its activation under control conditions than by PKA-dependent phosphorylation. Intriguingly, the RyR2-S4565R mutation was identified in an infant who died during sleep and not with exertion [10]. Furthermore, on the basis of experimental data, another mutation (RyR2-H29D) is suspected to cause arrhythmias both at rest and with exertion [11].

Similarly, although not yet tested experimentally, the novel mutation found in our patient caused VT both at rest and under exercise. In a retrospective cohort study of CPVT, in only one-fourth of symptomatic patients were cardiac events provoked by only normal wakeful activities [12].

Although it is unclear if in our patient the VT at rest was linked to adrenergic stimulation during sleep, the previously identified mutations RyR2-S4565R and RyR2-H29D at least suggest a mechanism that is potentially independent of sympathoadrenergic activation. Therefore, further investigation is needed to clarify this question.

The identification of mutations that possibly trigger the induction of ventricular arrhythmia without sympathoadrenergic stimulation makes it challenging to develop a regimen for patient-tailored treatments. General recommendations made by the European Society of Cardiology suggest lifestyle changes with avoidance of competitive sports, strenuous exercise, and stressful environments. Medical treatment should include beta-blockers in all patients with a clinical diagnosis of CPVT, based on the presence of documented spontaneous or stress-induced ventricular arrhythmias. Flecainide should be considered in addition to beta-blockers in patients who experience recurrent syncope or polymorphic/bidirectional VT while on beta-blockers [4].

ICD implantation is recommended for all patients with a diagnosis of CPVT who have survived cardiac arrest, recurrent syncope, or polymorphic/bidirectional VT despite optimal therapy. Finally, left cardiac sympathetic denervation may be considered in patients with a diagnosis of CPVT who experience recurrent syncope or polymorphic VTs [4].

Further evidence is needed to clarify if some genetic mutations result in mechanisms that trigger ventricular arrhythmia that are not linked to sympathoadrenergic stimulation. Supposing that some mutations may cause arrhythmias independent of sympathoadrenergic stimulation, the conventional recommendations have to be rethought, dependent on the respective mutation.

## Conclusions

We present a case of a patient with CPVT in both resting conditions and physical activity. The patient’s CPVT was associated with an as yet undescribed single point mutation in the RyR2 gene. This novel mutation may cause arrhythmias independent of sympathoadrenergic stimulation, but further research and evidence are needed to prove causality.

## Abbreviations

CASQ2: Calsequestrin
CPVT: Catecholaminergic polymorphic ventricular tachycardia
ICD: Implantable cardioverter defibrillator
ICU: Intensive care unit
PKA: Protein kinase A
RyR2: Ryanodine receptor 2
SR: Sarcoplasmic reticulum
VF: Ventricular fibrillation
VT: Ventricular tachycardia
